# Intravitreal itraconazole inhibits laser-induced choroidal neovascularization in rats

**DOI:** 10.1371/journal.pone.0180482

**Published:** 2017-06-30

**Authors:** Jeong Hun Bae, Ah Reum Hwang, Chan Yun Kim, Hyeong Gon Yu, Hyoung Jun Koh, Woo Ick Yang, Hae Ran Chang, Sung Chul Lee

**Affiliations:** 1 Medical Research Institute, Department of Ophthalmology, Kangbuk Samsung Hospital, Sungkyunkwan University School of Medicine, Seoul, Republic of Korea; 2 Department of Ophthalmology, Yonsei University College of Medicine, Seoul, Republic of Korea; 3 Department of Ophthalmology, Seoul National University College of Medicine, Seoul, Republic of Korea; 4 Department of Pathology, Yonsei University College of Medicine, Seoul, Republic of Korea; University of Florida, UNITED STATES

## Abstract

Choroidal neovascularization (CNV) is a major cause of severe visual loss in patients with age-related macular degeneration (AMD). Recently, itraconazole has shown potent and dose-dependent inhibition of tumor-associated angiogenesis. We evaluated the anti-angiogenic effect of itraconazole in a rat model of laser-induced CNV. After laser photocoagulation in each eye to cause CNV, right eyes were administered intravitreal injections of itraconazole; left eyes received balanced salt solution (BSS) as controls. On day 14 after laser induction, fluorescein angiography (FA) was used to assess abnormal vascular leakage. Flattened retinal pigment epithelium (RPE)-choroid tissue complex was stained with Alexa Fluor 594-conjugated isolectin B4 to measure the CNV area and volume. Vascular endothelial growth factor receptor 2 (VEGFR2) mRNA and protein expression was determined 1, 4, 7, and 14 days after intravitreal injection by quantitative RT-PCR or Western blot. VEGF levels were analyzed by enzyme-linked immunosorbent assay (ELISA). Intravitreal itraconazole significantly reduced leakage from CNV as assessed by FA and CNV area and volume on flat mounts compared with intravitreal BSS (p = 0.002 for CNV leakage, p<0.001 for CNV area and volume). Quantitative RT-PCR showed significantly lower expression of VEGFR2 mRNA in the RPE-choroid complexes of itraconazole-injected eyes than those of BSS-injected eyes on days 7 and 14 (p = 0.003 and p = 0.006). Western blots indicated that VEGFR2 was downregulated after itraconazole treatment. ELISA showed a significant difference in VEGF level between itraconazole-injected and BSS-injected eyes on days 7 and 14 (p = 0.04 and p = 0.001). Our study demonstrated that intravitreal itraconazole significantly inhibited the development of laser-induced CNV in rats. Itraconazole had anti-angiogenic activity along with the reduction of VEGFR2 and VEGF levels. Itraconazole may prove beneficial for treating CNV as an alternative or adjunct to other therapies.

## Introduction

Age-related macular degeneration (AMD) is the leading cause of blindness in older people in industrialized countries [1]. Severe vision loss in patients with AMD is commonly due to choroidal neovascularization (CNV) [2]. Although the CNV pathogenesis is not clearly understood, pathological angiogenesis mediated by growth factors, such as vascular endothelial growth factor (VEGF) and fibroblast growth factor, is important in CNV development. These factors induce activation of signaling pathways after binding receptors on endothelial cells. Several key signaling mediators including phospholipase C, phosphatidylinositol-3 kinase, and protein kinase C deliver angiogenic signals to stimulate endothelial cell proliferation, migration, and survival.

Currently available treatments for CNV include laser photocoagulation, photodynamic therapy, and intravitreal injection of anti-VEGF drugs. Although these treatments are helpful in reducing the risk of visual deterioration, they only improve vision in 6–33% of patients [3,4]. In addition, patients often exhibit AMD recurrence and require repeated treatments. Multiple intravitreal injections for months or years are associated with complications, such as cataract, retinal detachment, and endophthalmitis, and increase treatment costs [5]. To enhance the outcomes of CNV treatment and reduce patient costs, novel, alternative anti-angiogenic drugs are being investigated.

Itraconazole is a triazole antifungal drug that inhibits the enzyme lanosterol 14-α-demethylase, which is important in ergosterol synthesis in fungi. Previous studies reported that itraconazole has anti-angiogenic effects in *in vitro* assays and murine models [6,7]. The anti-angiogenic mechanism involves inhibiting the mammalian target of rapamycin (mTOR) pathway in human umbilical vein endothelial cells (HUVEC), hedgehog signaling in NIH-3T3 cells, and N-glycosylation in macrophages [8–10]. Itraconazole also inhibits activation of VEGF receptor 2 (VEGFR2), the receptor that is primarily responsible for angiogenesis [11]. Since itraconazole showed a dose-dependent inhibitory effect on endothelial cell proliferation, migration, and tube formation stimulated by VEGFR2-mediated signaling, itraconazole might be useful as an inhibitor of pathological angiogenesis. However, few studies have evaluated the effect of itraconazole in ocular neovascular disorders [12].

In this study, we investigated the anti-angiogenic effect of itraconazole in a rat model of laser-induced CNV, which is a widely used CNV model and is well-suited for assessing the inhibitory effects of intraocular drugs [13].

## Materials and methods

### Animals

Male Brown Norway rats weighing 200–250 g (Orient Bio Inc., Seongnam, Korea) were fed laboratory chow and water *ad libitum* and acclimated to the animal facilities at Kangbuk Samsung Hospital under a 12-hour on/off light cycle for one week. All experiments were designed and conducted in accordance with guidelines for animal use and ophthalmic research of the Association for Research in Vision and Ophthalmology and were approved by the Institutional Animal Care and Use Committee of Kangbuk Samsung Hospital, Sungkyunkwan University School of Medicine (Permit Number: 201503079).

### Induction of CNV

Before all procedures, animals were anesthetized with intraperitoneal zolazepam-tiletamine (Zoletil^®^, Virbac, Carros, France) at 30 mg/kg and xylazine hydrochloride at 10 mg/kg. Pupils were dilated with 0.5% tropicamide and 0.5% phenylephrine HCl. Proparacaine hydrochloride 0.5% was applied to anesthetize the cornea. Animals were positioned in front of a slit-lamp biomicroscope, and the fundus was visualized using a slide glass. A 532-nm Nd:YAG laser (Pattern Scan Laser, OptiMedica Corp., Santa Clara, CA, USA) was used to induce CNV by rupturing the Bruch membrane. The laser parameters were 60-μm spot size, 30-ms exposure, and 320-mW power. Six laser burns were placed concentrically, two disc diameters from the optic disc in both eyes of all animals. Subretinal bubbles indicated ruptured Bruch membranes. Eyes with vitreous or subretinal bleeding or no bubble formation were excluded.

### Preparation and intravitreal injection of itraconazole

Itraconazole 250 mg/25 ml (Sporanox^®^, Janssen Pharmaceuticals Inc., Buckinghamshire, UK) was diluted to a final concentration of 100 μg/ml under sterile conditions. Itraconazole concentrations used in this study were previously reported to be nontoxic [14]. Immediately after inducing CNV, intravitreal injection was performed in eyes with laser induction and normal control eyes. Topical proparacaine hydrochloride 0.5% was applied to the eye surface, and an initial hole was made with a 30-gauge needle 2.0 mm posterior to the limbus in the superotemporal quadrant. The eye was gently massaged with a cotton tip to remove prolapsed vitreous. A 32-gauge needle on a 50-μl Hamilton syringe was inserted into the mid-vitreous cavity through the initial hole at a 45° angle to the scleral surface to avoid injuring the lens with the needle [15]. Animals were given intravitreal injections of itraconazole 1 μg/10 μl in the right eye and 10 μl balanced salt solution (BSS) in the left eye. Before and immediately after injection, 0.5% moxifloxacin hydrochloride ophthalmic solution (Vigamox^®^, Alcon, Fort Worth, Texas, USA) was applied to the ocular surface.

### Leakage assessment and CNV grading

On day 14 after laser induction, 12 rats underwent fluorescein angiography (FA) with a Heidelberg Retina Angiograph (HRA; Heidelberg Engineering, Heidelberg, Germany) at a viewing angle of 50 degrees. For a wider field of view, a 20-diopter lens was attached to the fundus camera. For FA examinations, 0.3 ml of 10% sodium fluorescein (5 ml; Novartis Korea, Seoul, Korea) was administered intraperitoneally, and photographs were taken by a single trained operator using the Automatic Real Time (ART) module (Heidelberg Engineering, Heidelberg, Germany) at 30 seconds and 1, 2, 5, 10, and 15 minutes after dye injection. Angiograms at five-minute (±1 minute) phase were used for comparative analyses. Images were evaluated according to grading criteria [16] by two retinal specialists (JHB and SCL) in a masked fashion. CNV lesions were classified as grade 0 (no hyperfluorescence), grade I (hyperfluorescence without leakage), grade IIA (hyperfluorescence in early or mid-transit images and late leakage), or grade IIB (bright hyperfluorescence in transit images and late leakage beyond lesions). Grade IIB was defined as clinically significant. The proportions of grade IIB CNV lesions in itraconazole- and BSS-injected eyes were compared.

### Size measurement of CNV on choroidal mounts

On day 14 after inducing CNV, five rats were euthanized by cervical dislocation, and their eyes were enucleated and fixed with 10% formalin for 2 hours. The cornea and lens were removed, four radial cuts were made to flatten the tissue, and the neural retina was gently removed from the eyecup. Retinal pigment epithelium (RPE)-choroid-sclera complexes were washed with phosphate-buffered saline (PBS; 137 mM NaCl, 2.7 mM KCl, 8.1 mM Na_2_HPO_4_, 1.5 mM KH_2_PO_4_) and incubated in blocking solution (1% bovine serum albumin, 10% normal goat serum, 0.5% Triton X-100 in PBS) for 30 minutes. Alexa Fluor 594-conjugated isolectin B4 (1:100, 1 mg/ml solution, lectin from *Bandeiraea simplicifolia*, Invitrogen, Carlsbad, CA, USA) was applied to eyecups overnight at 4°C in blocking solution. After washing with PBS, eyecups were placed flat with the RPE-side facing up on microscope slides. Flat mounts were examined by fluorescence microscopy (IX71; Olympus, Tokyo, Japan), and CNV images were captured with a digital camera attached to the microscope. Under magnification of digital images, the borders of hyperfluorescent CNV lesions were manually outlined by a masked investigator (HGY), and the area of CNV lesions on flat mounts was measured. For the measurements of CNV volume, Z-stack images through the entire CNV lesion were taken at a constant intensity by a laser confocal microscope (LSM510 META; Carl Zeiss, Jena, Germany). Image analysis was performed using ImageJ software (National Institutes of Health, Bethesda, MD, USA), and the average area and volume of the CNV lesions were used for evaluation.

### Real-time quantitative polymerase chain reaction

On days 1, 4, 7, and 14 after intravitreal injection, RPE-choroid complexes were isolated from three eyes from each group at each time point and stored at -80°C until use. RT-qPCR was performed with the StepOnePlus real-time PCR system (Applied Biosystems, Foster City, CA, USA) as described elsewhere [17]. Total RNA was purified with RNeasy Mini Kits (Qiagen, Valencia, CA, USA), and cDNA was prepared using SuperScript III first-strand synthesis kits (Invitrogen, Carlsbad, CA, USA) with 0.5 μg total RNA according to the manufacturer's recommendations. Quantitative PCR used primers specific for rat VEGFR2 and β-actin with SYBR Premix Ex Taq (Takara Bio, Inc., Shiga, Japan). Primer sequences were rat VEGFR2 5′-CTC CAT CTT TTG GTG GGA TG-3′ (forward) and 5′-AGG CCA CAG ACT CCC TGC TTT TAC TG-3′ (reverse) and rat β-actin 5′-TGC CTG ACG GTC AGG TCA-3′ (forward) and 5′-CAG GAA GGA AGG CTG GAA G-3′ (reverse). All RT-qPCR experiments were performed in triplicate.

The housekeeping gene β-actin was an internal control, and VEGFR2 mRNA levels were normalized against β-actin levels. The StepOnePlus real-time PCR system was used to analyze data and obtain threshold cycle (CT) values according to the manufacturer's instructions. The ΔΔCT method was used to transform CT values into relative quantities with standard deviations [18].

### Western blot analysis

VEGFR2 expression was evaluated by Western blot. On days 1, 4, 7, and 14 after intravitreal injection, RPE-choroid complexes (three eyes per group at each time point) were homogenized with a bead beater at 4°C in cell lysis buffer (Cell Signaling Technology, Danvers, MA, USA). Samples were centrifuged at 14,000 rpm for 15 minutes at 4°C, and protein concentrations of the supernatants were measured with the Pierce™ Bicinchoninic Acid Protein Assay Kit (Thermo Fisher Scientific, Inc., Rockford, IL, USA). Soluble proteins (30 μg per sample) were boiled for 5 minutes and resolved by 10% SDS-PAGE. Proteins were electrotransferred to 0.45-μm-pore polyvinylidene fluoride membranes and blocked with 5% skim milk. Membranes were blotted overnight with primary antibodies diluted in 0.1% bovine serum albumin and 0.01% sodium azide in TBS-T for VEGFR2 (Novus Biologicals) and β-actin. After washing three times with TBS-T, blots were incubated with horseradish peroxidase-conjugated secondary antibody (Cell Signaling Technology) for 1 hour at room temperature. Blots were washed three times with TBS-T, and immunoreactive bands were visualized by enhanced chemiluminescence. Relative intensities of immunoreactive bands were measured after normalization for β-actin. Representative Western blots are shown after three independent experiments.

### Enzyme-linked immunosorbent assay

VEGF protein was analyzed in enucleated eyes via enzyme-linked immunosorbent assay (ELISA) 1, 4, 7, and 14 days after inducing CNV. The cornea and lens were removed, and the retina was separated from the eyecup. Retinas were homogenized in cell lysis buffer and incubated for 30 minutes on ice. Lysate was centrifuged at 14,000 rpm for 15 minutes at 4°C. Supernatants were assayed using commercially available ELISA kits against rat VEGF (RayBiotech Inc., Norcross, GA, USA) according to the manufacturer’s protocols. Total protein concentrations were determined using the Pierce™ BCA Protein Assay Kit (Thermo Fisher Scientific, Inc., Rockford, IL, USA) as the standard. VEGF concentrations were measured by ELISA in triplicate.

### Statistical analyses

All statistical analyses were performed with the statistical package PASW 18.0 (SPSS Inc., Chicago, IL, USA). All values are mean ± standard deviation. Comparative analyses of two values between groups were made with the unpaired *t*-test or the Mann-Whitney U test. FA image grades were compared with a chi-square test. *P* < 0.05 was considered significant.

## Results

### Inhibition of CNV formation by itraconazole

**Fig 1:**
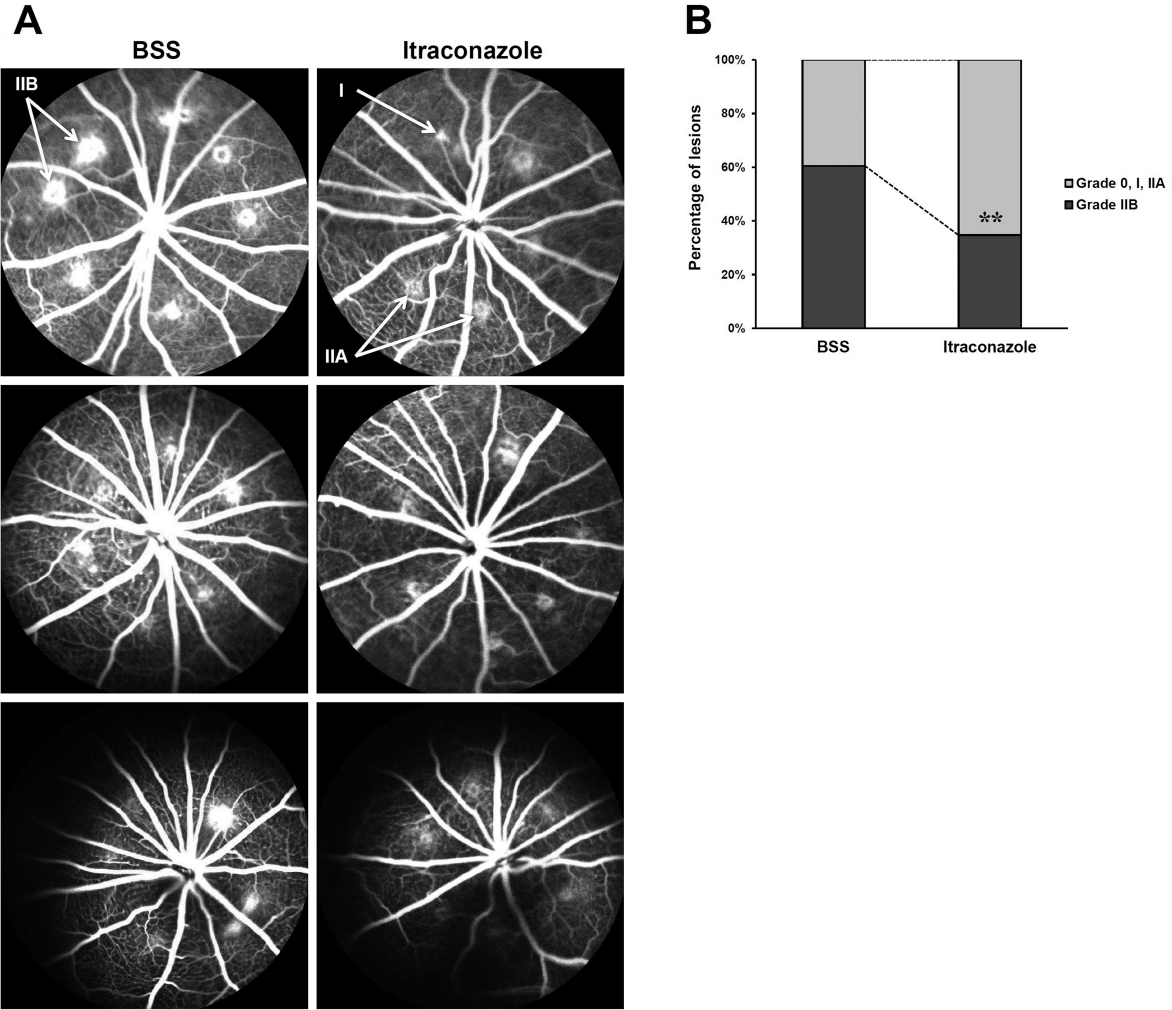
Representative fluorescein angiography (FA) of choroidal neovascularization (CNV) lesions. Six laser burns were introduced around the optic disc of each eye. Immediately after inducing CNV, either itraconazole or balanced salt solution (BSS) was injected intravitreally. On day 14, FA was conducted to assess leakage from CNV. (A) Midphase (5 ± 1 minutes) FA of eyes injected with itraconazole or BSS. Arrows, lesion grades of laser-induced CNV: grade I, grade IIA, or grade IIB. (B) Percentage of grade 0, I, IIA (defined as no-to-moderate leakage), and IIB (considered clinically significant) lesions in itraconazole-injected eyes (n = 12) and BSS-injected eyes (n = 12). ^**^p = 0.002.

To assess the inhibitory effect of itraconazole on CNV formation, FA and isolectin B4 staining of RPE-choroid complex flat mounts were conducted on day 14 after inducing CNV. FA showed that intravitreal itraconazole significantly reduced fluorescein leakage from CNV compared with intravitreal BSS (Fig 1A). The incidence of grade IIB CNV lesions was significantly lower in itraconazole-injected eyes (34.7%, 25 of 72 lesions) than in BSS-injected eyes (61.1%, 44 of 72 lesions) (p = 0.002, Fig 1B).

**Fig 2:**
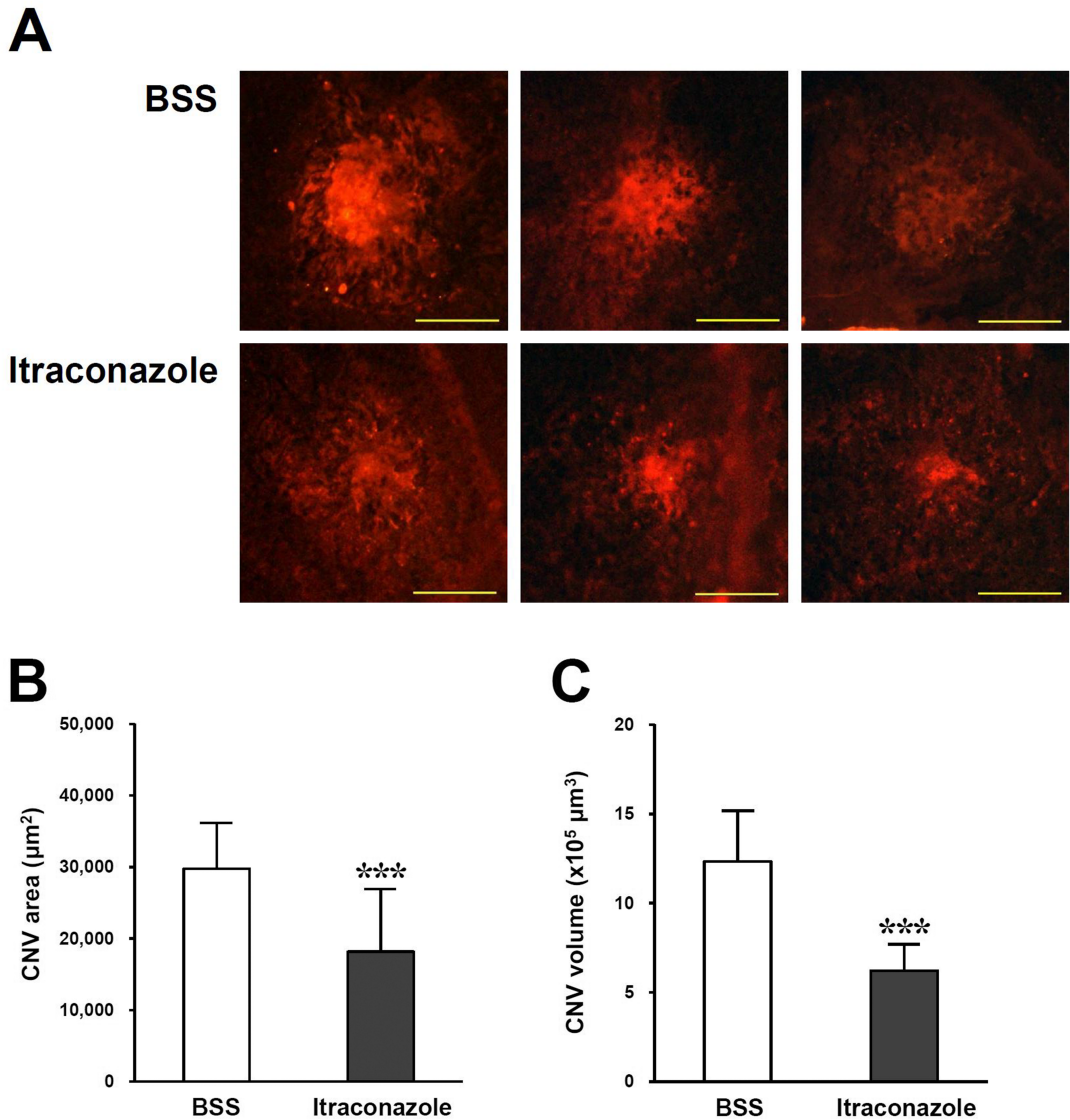
(A) Representative fluorescein microscopic photographs of flat mounts of the RPE-choroid complex stained with Alexa Fluor 594-conjugated isolectin B4 from *Bandeiraea simplicifolia* on day 14 after inducing CNV in itraconazole-injected and BSS-injected eyes. (B and C) Reduced CNV area and volume stained with isolectin B4 were significant in itraconazole-injected eyes compared with BSS-injected eyes. Values represent mean ± standard deviation (^***^p<0.001). Scale bar = 200 μm.

Fig 2A shows isolectin B4-stained endothelial cells for angiogenesis in flat mounts of the RPE-choroid complex. CNV lesions were smaller in the itraconazole group than in the BSS group. The mean CNV area was significantly reduced in itraconazole-injected eyes (18154 ± 8767 μm^2^, n = 30 lesions) compared with BSS-injected eyes (29762 ± 6376 μm^2^, n = 30 lesions; p<0.001, Fig 2B). Similarly, the mean CNV volume was also significantly decreased in itraconazole-injected eyes (1232 ± 284 x 10^3^ μm^3^, n = 18 lesions) compared with BSS-injected eyes (620 ± 151 x 10^3^ μm^3^, n = 18 lesions; p<0.001, Fig 2C).

These results suggest that intravitreal itraconazole effectively suppresses CNV development and reduces vascular leakage in rats.

### Reduction of VEGFR2 mRNA and protein after itraconazole treatment

**Fig 3:**
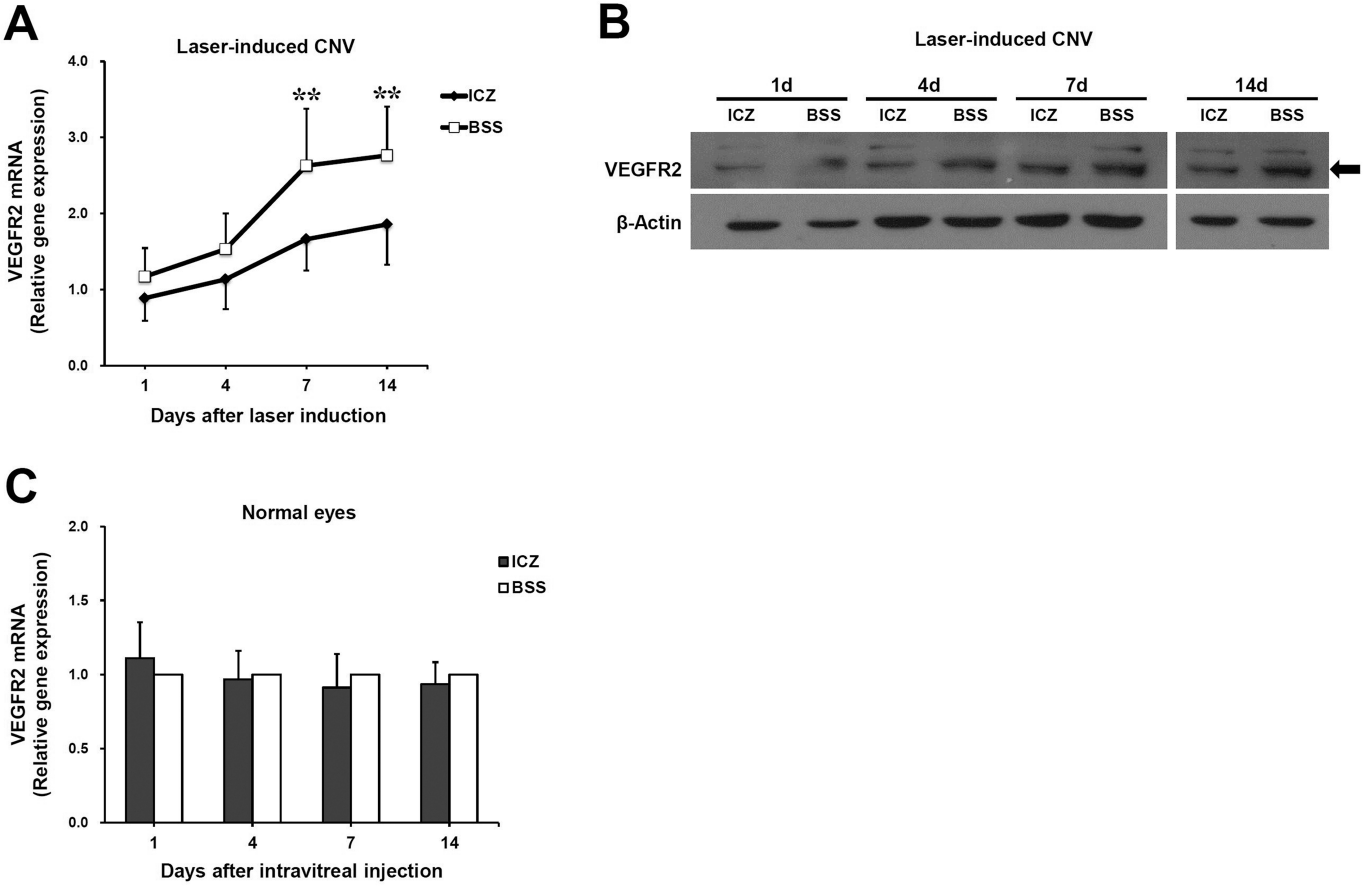
Inhibitory effect of itraconazole on VEGFR2 expression. (A) Quantitative analysis of VEGFR2 mRNA by RT-qPCR. After inducing CNV, either itraconazole or BSS was injected into the vitreous cavity of rat eyes. On days 1, 4, 7, and 14 after injection, the RPE-choroid complexes were separated and subjected to assay. VEGFR2 mRNA level increased gradually from day 1 to day 14. Relative VEGFR2 mRNA expression was significantly lower in itraconazole (ICZ)-injected eyes than in BSS-injected eyes on days 7 and 14 (^**^p = 0.003 and ^**^p = 0.006, respectively). (B) Western blots of the RPE-choroid complex on days 1, 4, 7, and 14 after laser induction. The density of the VEGFR2 bands, as indicated by a black arrow, decreased in ICZ-injected eyes compared to BSS-injected eyes at each time point. (C) RT-qPCR of RPE-choroid tissue in normal rat eyes injected either with ICZ or with BSS. The level of VEGFR2 mRNA in normal eyes was low and not significantly different between ICZ-injected and BSS-injected normal eyes. Values are mean ± standard deviation (n = 9 eyes per group at each time point). Relative gene expression indicates the ratio of VEGFR2 mRNA levels relative to those in BSS-injected normal eyes.

As VEGF-mediated signaling plays an important role in CNV development, we investigated the mRNA and protein levels of VEGFR2, a key receptor for pathological angiogenesis stimulated by VEGF. To determine whether itraconazole affected expression of VEGFR2 in a rat model of laser-induced CNV, RT-qPCR was performed for VEGFR2 mRNA in the RPE-choroid complex 1, 4, 7, and 14 days after laser induction. RT-qPCR showed that VEGFR2 mRNA increased gradually at each time point. The relative expression of VEGFR2 mRNA was significantly lower in itraconazole-injected eyes than in BSS-injected eyes on days 7 and 14 (p = 0.003 and p = 0.006, respectively; relative gene expression indicates the ratio of VEGFR2 mRNA levels relative to those in BSS-injected normal eyes, Fig 3A).

Similarly, Western blots on days 1, 4, 7, and 14 after inducing CNV indicated that itraconazole treatment prominently reduced the density of the VEGFR2 band in the RPE-choroid complex of rat eyes compared to BSS (Fig 3B). These results suggest that itraconazole might prevent the development of laser-induced CNV along with the reduction of VEGFR2 mRNA and protein.

To evaluate the effects of itraconazole on the expression of VEGFR2 in normal rat eyes, RT-qPCR was performed for VEGFR2 mRNA in the RPE-choroid complex 1, 4, and 7 days after intravitreal injection. The expression of VEGFR2 mRNA remained low in normal eyes, and there was no significant difference in the relative expression level of VEGFR2 mRNA between itraconazole-injected normal eyes and BSS-injected normal eyes at any time point (Fig 3C).

### Reduction of VEGF level after itraconazole treatment

**Fig 4:**
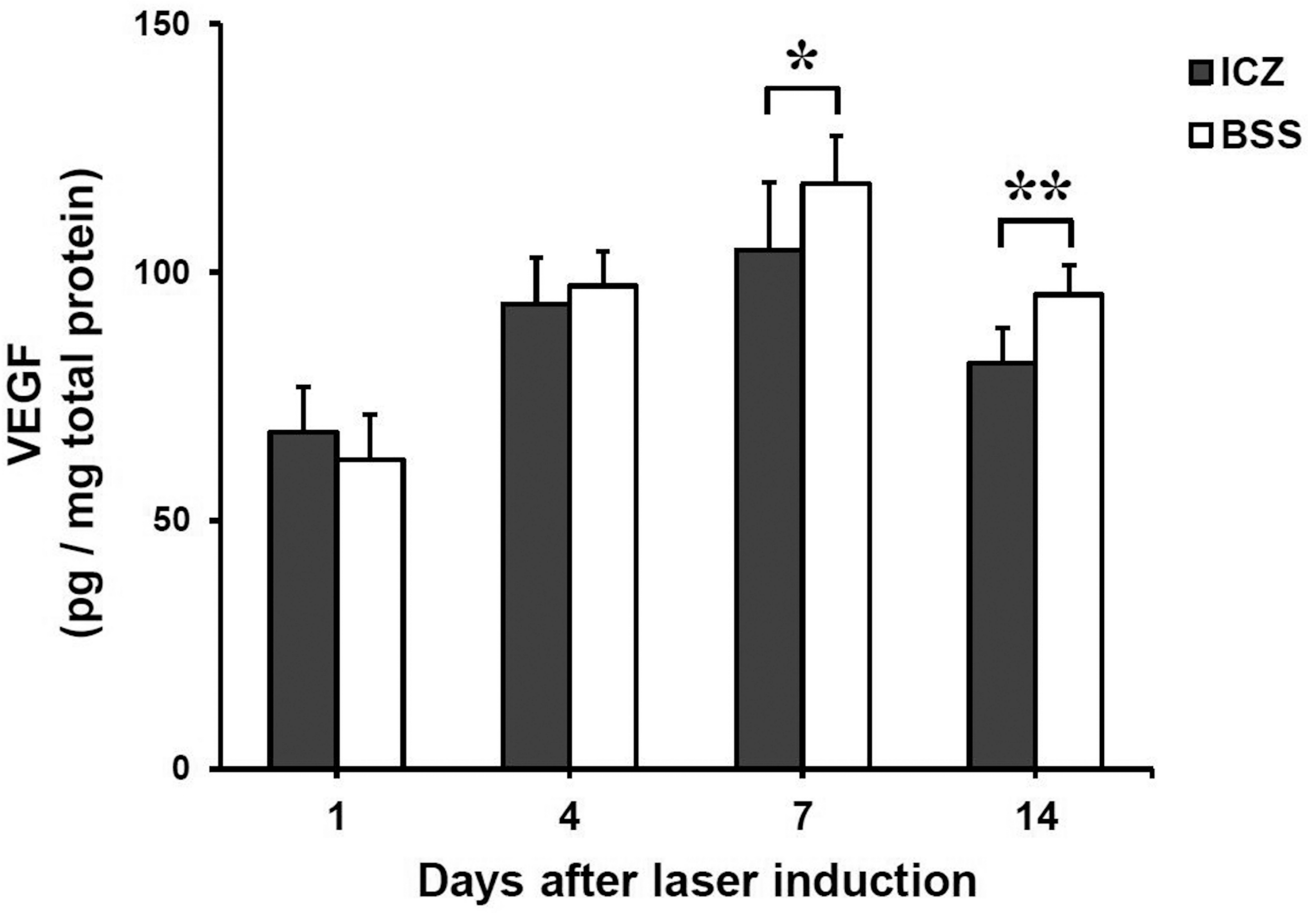
Effect of itraconazole on VEGF levels in laser-induced CNV. ELISA showed that the mean normalized VEGF levels in the retina increased markedly after laser induction. The highest level of VEGF was observed on day 7. There were significant differences in VEGF levels between itraconazole (ICZ)-injected eyes and BSS-injected eyes on days 7 and 14 (^*^p = 0.04 and ^**^p = 0.001, respectively). Values are mean ± standard deviation (n = 9 eyes per group at each time point).

Because itraconazole inhibited VEGFR2 expression, we further evaluated whether itraconazole affected the intraocular level of VEGF protein in laser-induced CNV. ELISA was performed with cleared lysates of retinal protein 1, 4, 7, and 14 days after inducing CNV. The mean normalized VEGF levels in the retina increased considerably after laser induction, with the highest VEGF level observed on day 7 in both groups. There were significant differences in retinal VEGF levels between itraconazole-injected eyes and BSS-injected eyes on days 7 and 14 (p = 0.04 and p = 0.001, respectively, Fig 4). Itraconazole treatment was associated with reduction of the retinal VEGF level in laser-induced CNV after 7 days.

## Discussion

In this study, we identified a novel effect of itraconazole as an anti-angiogenic agent in suppressing CNV development in rats. On day 14 after inducing CNV, intravitreal itraconazole significantly reduced fluorescein leakage from CNV and the vascular budding area on flat mounts of the RPE-choroid complex. Our results also demonstrated that VEGFR2 mRNA and protein levels in the RPE-choroid complex were reduced in laser-induced CNV after intravitreal itraconazole. However, the reduction of VEGFR2 after intravitreal itraconazole was not significant in normal rat eyes. This finding suggests that the inhibitory effect of itraconazole occurs selectively in pathological angiogenesis. To our knowledge, this is the first report demonstrating the effects of intravitreal itraconazole in a rat model of CNV.

Several studies have reported the potent anti-angiogenic activity of itraconazole, and multiple phase II human clinical trials are currently ongoing to study itraconazole as a cancer treatment [6–9,19]. A recent *in vivo* study reported that oral itraconazole inhibited the growth of primary xenografts of human non-small cell lung cancer [7]. In addition, a noncomparative randomized phase II clinical trial suggested that oral itraconazole had modest antitumor activity by downregulating hedgehog signaling in advanced prostate cancer [20]. Because of its good safety profile as an antifungal drug and its relatively high serum levels, itraconazole is a candidate anticancer drug. Given its promising results as an anticancer drug, itraconazole may have efficacy in treating ocular pathological angiogenesis such as CNV. In our study, mean CNV area was reduced by 39% in itraconazole-injected eyes compared to BSS-injected eyes, which was similar to previous similar studies with other drugs. Recently, intravitreal treatment of triamcinolone acetonide was reported to reduce the CNV area by 41.8% compared to vehicle treatment in laser-induced CNV in mice, and eyes with intravitreal VEGF-TRAP showed a significant 29.9% reduction in CNV area compared to controls in a mouse model of laser-induced CNV [21,22].

The mechanisms of CNV pathogenesis are complicated and not fully understood. Multiple signaling pathways are involved in CNV development. Chronic oxidative stress in the RPE and photoreceptors increases HIF-1 levels, which activate the gene expression of angiogenic growth factors, such as VEGF and platelet-derived growth factor [23,24]. However, increased VEGF expression in RPE cells is not sufficient to cause CNV without abnormalities or defects in Bruch’s membrane [25]. In addition to the angiogenic factors associated with CNV, the extracellular matrix and surrounding cells facilitate survival and stabilize endothelial cells [26]. Animal studies showed that inflammatory cells such as macrophages play a critical role in choroidal angiogenesis through the recruitment and activation in CNV lesions to release pro-angiogenic factors [27,28]. Thus, considering the various pathogenic factors involved in CNV, therapies targeting multiple participants in CNV pathogenesis would provide benefits beyond a single-target therapy.

Itraconazole, like other azole antifungal drugs, inhibits lanosterol demethylation to ergosterol in fungi and to cholesterol in humans. Cellular cholesterol trafficking activates the mTOR pathway regulating multiple environmental signals required for cell growth and proliferation [8,29]. Therefore, itraconazole could interrupt endothelial cell proliferation by lowering cholesterol levels and inhibiting mTOR [30]. However, inhibiting lanosterol 14-α-demethylase alone is not sufficient to explain itraconazole’s anti-angiogenic properties because other azoles do not inhibit endothelial cell proliferation [6,19]. In some studies, itraconazole potently and selectively inhibited multiple key regulators of tumor angiogenesis in murine models and *in vitro* assays using HUVECs and human dermal microvascular endothelial cells (HMEC-1) [7,31]. Furthermore, itraconazole potently inhibits the hedgehog signaling pathway, which is implicated in angiogenesis [9,20]. In fact, blockade of the mTOR pathway or hedgehog signaling pathway has proven to be effective in reducing CNV in rodent models [32,33]. Thus, the inhibition of those pathways via itraconazole treatment may show efficacy in preventing CNV, but further investigation is needed to determine its effect.

Itraconazole was reported to inhibit VEGFR2 glycosylation and autophosphorylation and VEGF binding to VEGFR2, which might be important in inhibiting angiogenesis *in vivo* [11]. VEGF is critical for CNV development, and preclinical and clinical data suggest that the VEGF pathway may be a primary therapeutic target for CNV treatment [34]. Blocking VEGF receptors can provide another way to antagonize VEGF and can potentially block multiple VEGF subtypes. Of the VEGF receptors, VEGFR2 is involved in almost all VEGF-mediated responses in pathological angiogenesis [35–37]. Thus, inhibiting VEGFR2 expression could result in anti-VEGF activity by preventing VEGF-mediated signaling, leading to suppression of laser-induced CNV in rats. In our study, VEGFR2 was markedly expressed in RPE-choroid tissue 7 days after laser induction, and the inhibitory effect of itraconazole was prominent in the late period when CNV typically began to form; itraconazole effectively prevented the development of CNV. However, the reduction of VEGFR2 level after itraconazole treatment might result from fewer VEGFR2-expressing cells in RPE-choroid tissue, rather than from lower VEGFR2 expression on endothelial cells. Our data could not confirm that VEGFR2 expression on endothelial cells would be suppressed by itraconazole treatment. Considering the complicated pathogenesis of CNV, the anti-angiogenic effects of itraconazole might have a common downstream target *in vivo*. Thus, how closely the effects are associated with one another and how potently they inhibit angiogenesis with itraconazole treatment should be investigated.

Anti-VEGF agents such as ranibizumab and aflibercept inhibit intraocular VEGF activity and are promising treatments for CNV from AMD. Given the multifactorial pathogenesis of AMD, however, a single anti-VEGF agent may not always be effective and would require monthly or bimonthly injections. The lack of therapeutic options for CNV and limitations associated with anti-VEGF antibody therapies have led to the search for other anti-angiogenic agents. Itraconazole is a well-tolerated and orally bioavailable drug that has a well-established safety profile. Targeting multiple pathways might enhance the efficacy of itraconazole as an anti-angiogenic agent. Therefore, itraconazole is a novel candidate for an alternative treatment for CNV.

In this study, we evaluated the ability of itraconazole to prevent CNV development when administered shortly after laser induction. Many therapeutic modalities have been evaluated in preventing laser-induced CNV [38–40]. However, patients with AMD who require treatment have an established disease, and efficacy in a preventive model of CNV does not necessarily correspond to the human disease. The treatment model for established CNV is more suitable for assessing the efficacy of drug therapies. Thus, further studies are warranted to determine the efficacy and proper dosage of intraocular itraconazole for treating CNV.

In summary, our study demonstrated that intravitreal itraconazole significantly inhibited the development of laser-induced CNV in rats. Itraconazole had anti-angiogenic effects along with the reduction of VEGFR2 and VEGF levels. Itraconazole may prove beneficial for treating CNV as an alternative or adjunct to other therapies.

## Supporting information

S1 Fig Original uncropped Western blots of the RPE-choroid complex on days 1, 4, 7, and 14 after laser induction. (TIF)
