# Supplementary figures and images for: Intravitreal itraconazole inhibits laser-induced choroidal neovascularization in rats
Source: PLoS One. 2017 Jun 30;12(6):e0180482. doi: 10.1371/journal.pone.0180482 (PMC5493406; doi:10.1371/journal.pone.0180482)

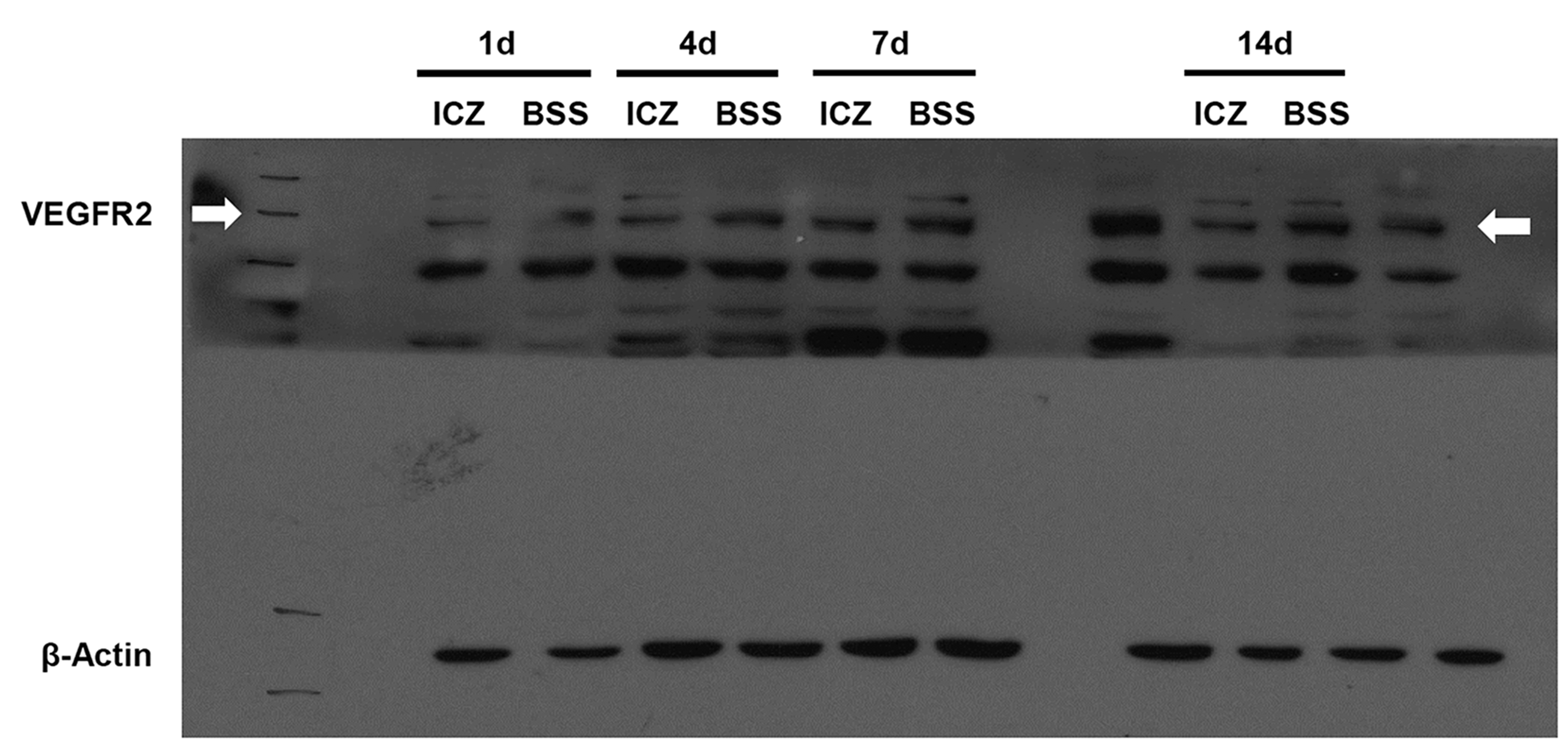

Supplement: S1 Fig — (TIF) [file pone.0180482.s001.tif]
